# Personality traits and polymorphisms of genes coding neurotransmitter receptors or transporters: review of single gene and genome-wide association studies

**DOI:** 10.1186/s12991-021-00328-4

**Published:** 2021-01-22

**Authors:** Szymon Zmorzyński, Wojciech Styk, Waldemar Klinkosz, Justyna Iskra, Agata Anna Filip

**Affiliations:** 1 Department of Cancer Genetics With Cytogenetic Laboratory, Medical University of Lublin, Lublin, Poland; 2 Institute of Psychology, The John Paul II Catholic University of Lublin, Lublin, Poland; 3 Institute of Psychology, Cardinal Stefan Wyszynski University, Warsaw, Poland

**Keywords:** DNA polymorphisms, Personality traits, GABA receptor-coding genes, Cannabinoid, Opioid receptor-coding genes, Dopaminergic receptor genes, Serotonin transporter genes, Neurotransmitter receptors, Neurotransmitter transporters

## Abstract

**Background:**

The most popular tool used for measuring personality traits is the Five-Factor Model (FFM). It includes neuroticism, extraversion, openness, agreeableness and conscientiousness. Many studies indicated the association of genes encoding neurotransmitter receptors/transporters with personality traits. The relationship connecting polymorphic DNA sequences and FFM features has been described in the case of genes encoding receptors of cannabinoid and dopaminergic systems. Moreover, dopaminergic system receives inputs from other neurotransmitters, like GABAergic or serotoninergic systems.

**Methods:**

We searched PubMed Central (PMC), Science Direct, Scopus, Cochrane Library, Web of Science and EBSCO databases from their inception to November 19, 2020, to identify original studies, as well as peer-reviewed studies examining the FFM and its association with gene polymorphisms affecting the neurotransmitter functions in central nervous system.

**Results:**

Serotonin neurons modulate dopamine function. In gene encoding serotonin transporter protein, *SLC6A4*, was found polymorphism, which was correlated with openness to experience (in Sweden population), and high scores of neuroticism and low levels of agreeableness (in Caucasian population). The genome-wide association studies (GWASs) found an association of 5q34-q35, 3p24, 3q13 regions with higher scores of neuroticism, extraversion and agreeableness. However, the results for chromosome 3 regions are inconsistent, which was shown in our review paper.

**Conclusions:**

GWASs on polymorphisms are being continued in order to determine and further understand the relationship between the changes in DNA and personality traits.

## Introduction

The principal variables used to describe personality are features that are also known as personality dimensions. The existence of these features can be inferred by observing human behavior and emotional reactions [1, 2].

American researchers Paul T. Costa and Robert R. McCrae are the authors of the most commonly used tools for measuring personality traits [1]. These are the Revised Neuroticism Extraversion Openness Personality Inventory (NEO-PI-R) and NEO Five-Factor Inventory questionnaires (NEO-FFI) [1]. The NEO-PI-R is shorter version of NEO-FFI [2]. Both questionnaires are used to assess the five major dimensions of personality traits, which are neuroticism, extraversion, openness, agreeableness and conscientiousness [2]. These five dimensions have been linked to emotional stability, active motivation, as well as cognition [3, 4].

Based on research, which was conducted in 50 countries, FFM has gained international recognition and is treated as a universal personality structure, common to people from different cultures and language groups [5]. The results of studies, which were conducted on twins and within families suggested, that personality traits are considerably determined genetically [6–8]. This means that the components of FFM are characterized via a high degree of heritability (40–60%)[7, 9]. Openness to experience is the feature with the highest index of inheritance at 61% [7]. Genome-wide association study (GWAS) of personality traits showed higher values of heritability for conscientiousness (30%), extraversion (35%) and neuroticism (25%) [10]. Nevertheless, studies on human personality are not only limited to five dimensions, but additionally include other factors such as hereditary and environmental influence [11]. Studies on twins indicated that genetic inheritance explains 73% of extraversion variability [8]. Lo et al. in meta-analysis of GWAS identified genomic loci, significantly associated with personality traits [9]. Of these genome-wide regions extraversion was associated with 12q23.3 containing *WSCD2* gene expressed in neurons, neuroticism with gene variants located on chromosome 8p23.1 and 22q13.2 [9].

Polymorphism defines the common differences that occur in DNA sequence of a population. These most often concern single nucleotides, and are known as single nucleotide polymorphisms (SNPs). They can occur in different DNA regions and may affect the functions of various proteins, including those synthesized by neurons. In turn, changes in the central nervous system (CNS) might be associated with personality traits. Neurotransmitter receptors/transporters in GABAergic or dopaminergic systems affect inter-neuronal signal transmission [12]. The numerous genetic studies examined psychiatric diseases, while in the field of personality traits relatively less work [13] has been done. The aim of this systemic review is to summarize data assessing the genetic polymorphisms of genes encoding neurotransmitter receptors or transmitters, and their relationship with FFM. Research data are currently few and in many cases conflicting due to small sample sizes or studies within different populations. Possible genetic factors associated with personality dimensions are characterized below.

## Methods

We followed the Preferred Reporting Items for Systemic Review and Meta-Analyses (PRISMA) indications in the study indication and selection [14]. We searched PubMed Central (PMC), Science Direct, Scopus, Cochrane Library, Web of Science and EBSCO databases from their inception to November 19, 2020, to identify original studies, as well as peer-reviewed studies examining the significance or identification of gene polymorphisms associated with neurotransmitter functions in central nervous system. The PubMed search was as follows: (polymorphism* [title/abstract] OR genome-wide [title/abstract] OR genome wide [title/abstract]) AND (personality [title/abstract] OR five-factor [title/abstract] OR five factor [title/abstract] OR big five [title/abstract]), and was adapted according to each database’s needs. The list of located papers was examined and cross-references for further relevant literature. We included single-gene studies, as well as GWAS focused on association of gene polymorphisms and personality traits in various populations (among males and females). Moreover, we included manuscripts written in English and published in any year. We excluded studies in which polymorphisms were analyzed in affected individuals by psychiatric disorders, neurodegenerative disorders, and addicted subjects.

## Results and discussion

In PubMed Central, on November 19, 2020, the above search produced 384 records, on Science Direct—293 records, on Scopus—1537 records, on Cochrane Library—682 records, on Web of Science—252 records. The records were reviewed to detect undefined literature. The analyzed gene studies have been dominated by single-gene candidates involved in neurotransmitter systems, for example dopamine, serotonin that mediate the effects of selected, mostly psychoactive drugs [13]. Often the results of these studies are inconsistent and inconclusive [15]. In our review analysis we have focused on the major findings of original researches and meta-analyses of the most studied gene polymorphisms in the field of FFM.

### GABA receptor-coding genes

Gamma-aminobutyric acid (GABA) is a neurotransmitter in the CNS that belongs to the group of inhibitory amino acids [16]. It affects the activity of pyramidal neurons that are essential for cognition [17]. Through its receptors, GABA inhibits the hypothalamic–pituitary–adrenal gland axis, which is involved in the response to stress [18]. GABA receptors belong to three different classes: GABA_A_, GABA_B_ and GABA_C_. Class A receptors are the most prevalent in CNS [19].

The GABA_A_ receptor subunits are encoded by different genes and have been grouped into seven distinct classes: α, β, γ, δ, ε, π and θ [20]. The first three classes contain a lot of isoforms, including GABA_A_α6, which is coded by the *GABRA6* gene (*locus* 5q34). SNP (rs3219151) at 1521 nucleotide (1521 T > C; T/C-allele) has been described in 3′-untranslated region (3′-UTR) of *GABRA6* gene [21]. Arias and co-workers in the study of 937 subjects found association of high scores of harm avoidance personalities with T-allele [21]. Individuals with CC genotype scored significantly lower extraversion levels compared to heterozygotes or homozygotes for the T-allele [22].

Although single-gene study by Uhart et al. found as association of *GABRA6* gene polymorphism with personality trait, GWAS did not confirm it [10, 22–25]. The study of Uhart and co-workers was carried out on a small sample size—56 cases varied in terms of race (*N* = 40 Caucasian, *N* = 11 African American, *N* = 5 Asian) [22]. The GWAS results reported by Pilia et al. from 6148 Sardinians, confirmed the association of 5q34 region (without *GABRA6* gene) with higher neuroticism scores [23]. In this *locus* only one SNP (rs1421989) of *TENM2* gene was significant. The Pilia’s GWAS was replicated by The New England Centenarian Study [26]. *GABRA6* gene polymorphism is located in the non-coding region and does not seem to affect receptor functions [22]. However, this gene is organized into a cluster with other genes (*GABRA1*, *GABRB2*, *GABRG2*) and its SNP may be in linkage disequilibrium (LD) with polymorphisms located in close proximity, e.g., *TENM1* and/or *DRD1* genes, which may give a non-directive association with personality traits [22].

Kim et al. found association of 5q35 containing dopamine receptor D1 (*DRD1)* gene with neuroticism higher scores [27]. Moreover, they were associated with premature mortality, range of negative emotional states and psychiatric disorders [28–30]. The studies among twins suggest that about 40% of the neuroticism variance is heritable, of which 15–37% is explained by SNPs [24, 31]. However, GWAS meta-analysis did not find an association of loci on chromosome 5 with neuroticism [32].

### Cannabinoid/opioid receptor-coding genes

The cannabinoid system interacts with dopaminergic and opioid system [33, 34]. Cannabinoid receptors 1 (CB1) are encoded by the *CNR1* gene (*locus* 6q14-15) and are found in various areas of the brain, as well as in tissues located outside CNS [35]. CB1 receptors modulate the action of dopamine neurotransmitter in the brain regions that form the rewarding effects system [36]. In the *CNR1* gene, there are SNPs and simple sequence length polymorphism (SSLP). SSLP is associated with the occurrence of multiple repeats of the trinucleotide microsatellite sequences—(ATT)_n_ [37, 38]. Juhasz et al. showed a significant correlation between *CNR1* gene polymorphisms and the level of neuroticism and agreeableness [39]. Two haplotypes were tested in this gene—the first included SSLP, as well as SNPs in 3′-UTR region—rs806366 (T>C), rs806368 (T>C), rs12720071 (A>G), rs4707436 (G>A), in intron—rs806369 (C>T) and in exon—rs1049353 (G>A). The second haplotype, also included SSLP and SNPs present in introns—rs2023239 (T>C), rs1535255 (T>G), rs806379 (A>T). In the study by Juhasz et al. (*N* = 1269), there was a statistically significant correlation of the first haplotype with reduced level of neuroticism [39]. In turn, the second haplotype was associated with higher level of neuroticism and lower level of agreeableness [39]. The study held by Yao et al. showed significant relationship of *CNR1* variants with extraversion [40]. Furthermore, they found association of *CNR1* polymorphisms with conscientiousness, agreeableness and openness [40].

Mu-opioid receptors (MOPRs) are widely distributed in brain neurons and their activation is mainly involved in the neurobiology of pain [41]. *NR3C1* gene (*locus* 5q31.3) encodes glucocorticoid receptor 1, which may act as a regulator of MOPRs transcription. The most common SNP (rs1799971) of *NR3C1* is present in exon 1, in the form of 118A>G [42]. Zhang et al. reported reduced MOPR expression in cells with G-allele [43]. Pecina et al. in the study of 50 right-handed, healthy non-smoking subjects, found association of G-allele with higher neuroticism scores [44]. Montag and co-workers found relationship between higher neuroticism scores and the presence of G-allele [45].

Kim et al. in GWAS of 1089 participants found association between 6q21 *locus* with *NKAIN2* gene and higher scores of extraversion [27]. This *locus* is in close proximity of *CNR1* gene. However, GWAS analyses did not confirm the relationship of *CNR1* and *NR3C1 *loci with personality traits [10, 23–25].

### Dopaminergic receptors and their genes

The dopaminergic D receptors (DRDs) are involved in intracellular signal transduction [46]. The SNP (rs6280) within exon 1 of *DRD3* gene (*locus* 3q13) results in substitution of serine (T-allele) to glycine (C-allele) at amino acid 9 in the N-extracellular domain of the receptor [47]. TT and TC genotypes had significantly higher scores of neuroticism in comparison to CC homozygote [48]. In contrast, Schlosser et al. did not find association of *DRD3* gene polymorphism with personality traits [49].

The cross-talk between dopamine and oxytocin pathways has been described [50]. This interaction is modulated by oxytocin receptor (OXTR), which is encoded by *OXTR* gene (*locus* 3p25.3). A common SNP (rs53576) in intron 3 (G-allele) might be associated with higher levels of neuroticism [51].

In GWAS analysis, Kim et al. found strongest associations of 3p24 and 3q13 loci (without *DRD3* gene) with extraversion and agreeableness, respectively [27]. 3p24 region containing *HMGB1P5* gene is in LD with *OXTR* gene. Moreover, GWAS has shown association of 3q13.13 region (without *DRD3* gene *locus*) with neuroticism [32].

However, other GWAS did not show relationship of these loci with FFM traits [9, 10, 25].

### Serotonin transporter genes

Associations between polymorphisms of serotonin transporter genes and personality traits have been reported. For example, neuroticism has been linked to a functional polymorphism in *SLC6A4* gene (*locus* 17q11.1-17q12) [52]. It encodes a serotonin transporter protein that modulates serotoninergic neurotransmission [52]. It plays a major role in the mechanism of serotonin synaptic recirculation [52]. The *SLC6A4* gene’s promoter sequence contains a polymorphism due to a variable number of tandem repeats (VNTRs). This change is referred as serotonin transporter linked polymorphic region (5-HTTLPR), and it occurs in the form of either an insertion (L-long allele) or a deletion (S-short allele) of 44 base pairs in the promoter sequence [53]. The long and short alleles are associated with high and low expression levels of *SLC6A4* gene, respectively [54]. Correlations between 5-HTTLPR variant and FFM features were analyzed, with results portraying a relationship between *SLC6A4* gene polymorphism and neuroticism [55, 56]. Carriers of S allele have a significantly higher level of neuroticism and a notably lower level of agreeableness [57]. It was discovered that children between the ages of 9–15 years with SS genotype had significantly higher levels of neuroticism and lower levels of openness to experience, agreeableness and conscientiousness [58]. Rahman et al. conducted studies on a population of adults in Sweden (*N* = 3112) and found a correlation between 5-HTTLPR polymorphism and FFM personality traits [59]. They showed, that only the openness to experience was significantly related to this polymorphism. In addition, they observed elevated, but not statistically significant, levels of neuroticism in men with the S allele [59]. However, meta-analyses of many studies involving polymorphisms of *SLC6A4* have shown that genetic variants of this gene are not consistently associated with neuroticism [60, 61].

Another gene in serotoninergic system, *SLC18A1* (*locus* 8p21.3), encodes the vesicular monoamine transporter 1 (VMAT1), which is involved in the uptake of serotonin, dopamine, norepinephrine into synaptic vesicles [62, 63] A non-synonymous SNP (rs1390938, Thr136Ile) affects anxiety associated with personality traits, such as neuroticism [64]. This variant might contribute to the quantitative differences of anxiety as unique personality trait [64].

Meta-analysis of Lo et al. and GWAS by Bae et al. found association of 8p23.1 region (in close proximity of *SLC18A1* gene) with high levels extraversion and low levels of neuroticism, and 17q13.2 *locus* (in close proximity of *SLC6A4* gene) with neuroticism, respectively [9, 10]. Other GWASs did not find relationship between FFM traits and polymorphisms of serotonin transporter genes [60, 61].

## Summary and conclusions

This systematic review focused on the possible relationships between the polymorphisms of selected genes and personality traits. Psychology of individual differences has several models describing personality; currently, researchers have paid close attention to identification of genes associated with FFM. These studies have been conducted in two ways, with some including the scanning of the entire human genome, whilst others involved the analysis of single genes [9, 10, 24, 25]. The interrelationships between different genes or between polymorphic alleles of the same gene and their effect on the characteristics of FFM were analyzed. The literature on this subject included only few studies and in many cases the results were inconsistent or even mutually exclusive. GWAS showed strong relationship of SNP or chromosomal loci with FFM, for example it was found an association of 5q34-q35, 3p24, 3q13 regions with higher scores of neuroticism, extraversion and agreeableness [23, 27]. However, the results for chromosome 3 regions are inconsistent [9, 10, 25]. Moreover, one of the studies found an association of *GABRA6* gene polymorphism with big-five model, but GWAS analysis on a larger cohort did not confirm this relationship [22, 26]. The chromosomal regions described as strongly associated with FFM in some GWAS were not always strongly associated with personality dimensions in other studies. The difficulty in the replication of GWASs is due to population stratification (taking into account ethnic origin, race, age group and number of individuals recruited to the study), selective reporting of obtained results, as well as omission of linkage disequilibrium in monogenic studies [65]. It is possible, that molecular mechanisms, besides functional SNPs, might influence the gene expression. For example, microRNA molecules may be associated with FFM traits. Further research studies are necessary to determine gene polymorphisms, which impact the values of certain personality traits.

Until now, personality traits have been defined using self-report questionnaires. The use of new techniques, such as next-generation sequencing, gives many opportunities for new discoveries enabling the search and analysis of relationships between genes and personality traits, as well as a revision of current views. Further studies, taking into account epigenetic factors, are necessary in this field.

## Data Availability

Not applicable.
